# Desirable Features of an Interdisciplinary Handoff

**DOI:** 10.2196/18914

**Published:** 2020-05-22

**Authors:** Anupam Ashutosh Sule, Dean Caputo, Jaskaren Gohal, Doug Dascenzo

**Affiliations:** 1 Department of Internal Medicine St Joseph Mercy Oakland Pontiac, MI United States; 2 Department of Nursing St Joseph Mercy Oakland Pontiac, MI United States

**Keywords:** handoff, transition, sign-out, electronic, interdisciplinary, interprofessional, communication, patient safety

## Abstract

Failure of communication of critical information during handoffs is one of the leading causes of medical errors, resulting in serious, yet preventable, adverse events in hospitals across the United States. Recent studies have shown that a majority of these errors occur during patient handoffs, with notable communication gaps in interdisciplinary handoffs. We suggest some features that would improve the handoff usability and effectiveness for interdisciplinary medical and nursing teams while potentially improving patient safety.

## The Challenge

The landmark publication “To Err is Human” cast a spotlight on the devastating effects of adverse events and identified communication failures as one of the root causes of human deaths due to medical errors [1]. The follow-up report, “Preventing Medication Errors,” estimated that 1.5 million preventable adverse events occur each year in the United States, with hospital-based errors costing US $3.5 billion per year [2]. Multiple studies have reported communication failure, particularly during handoffs, as the leading cause of preventable adverse events in hospitals [3-5]. The common errors during handoffs include the exclusion of critical information and the transfer of incorrect information [6].

Patient handoffs are an integral part of health care. We believe that future handoffs should have the following features.

## Interdisciplinary Communication Tools

Physicians, nurses, pharmacists, and unlicensed assistive personnel that may be involved in a patient’s care often function independently and communicate inadequately [7]. Coordination between various treatments and interventions is critical for preventing errors and fragmentation of care. Tools designed to support interdisciplinary communication and collaboration were found to result in more positive patient care outcomes [7]. Nurses who were given access to view a computerized patient handoff app that was developed for physicians found that the app was a reliable and timely source of information on patient status and plans for treatment or discharge [8]. Moreover, the access to this app helped the nurses develop effective care plans and increased their work satisfaction [8]. Since the shift time for each health care practitioner is different, making an interdisciplinary handoff is an arduous task. We believe that an interdisciplinary handoff tool will enhance care team collaboration and communication, thus breaking the current “siloed” discipline-specific handoff approach.

Handoffs written by any health care practitioner should be visible to all practitioners of any health care discipline in the care team.

## Standardized Communication Tools

Implementation of a standardized Inpatient Settings Accelerating Safe Sign-outs program resulted in a 23% relative reduction in the overall error rates in 6 out of 9 hospitals, without impeding the workflow [9]. The Situation, Background, Assessment, and Recommendation (SBAR) tool was developed in 2006 and has been widely adopted in nursing practice [10]. Although SBAR is useful for reporting short messages of critical patient information in an organized manner, a systematic review of 95 articles revealed that despite the well-known adverse consequences of inadequate nursing handoffs, very little research had been performed to identify the best practices [11]. The standardization of data collection has been shown to improve communication and reduce errors [12-14].

While there is undoubtedly a need for the implementation of a standardized handoff tool, there are several challenges and limitations that need to be considered [15]. There is considerable variability across institutions regarding preferred methods and formats for verbal and written handoffs [16]. Handoff practices are usually so deeply embedded in a given institution that efforts at improving them necessitate a transformational change of the entire institution’s culture [15,16].

More research studies need to be undertaken to identify the best practices and to standardize nursing handoffs across the health care system. Every health care institute should at least develop its own standardized nursing handoff protocol until a health care system-wide standardized handoff is adopted.

## Formal Handoff Education for Health Care Professionals

In 2018, the Accreditation Council for Graduate Medical Education (ACGME) published the Clinical Learning Environment Review (CLER) National Report of Findings in which residents, fellows, and nurses expressed that communication during care transitions was often incomplete or inaccurate [3]. Various teaching styles (eg, in-person didactic sessions, simulated clinical scenarios, video- and web-based teaching modules) can be used to teach a handoff curriculum [17]. The ACGME requires residency training programs to provide formal instruction on handoffs and to monitor handoff quality [6]. However, despite these requirements and patient safety concerns, well-established handoff curricula and validated tools to observe and assess the handoff skills of medical and nursing trainees are lacking [18]. The CLER report indicated that a standardized organization-wide approach for training and managing transition of care is uncommon across CLEs [3]. Developing an effective handoff curriculum proves challenging because of the need for standardized protocols, faculty education, cultural resistance to change, and diverse institutional factors.

Nursing and clinician educators at each institute should develop a curriculum to teach and reinforce the use of the handoff, which can be accepted as the institutional standard.

## Housed Within the Electronic Health Record

Chui et al [19] noted that the handoff practices in pharmacies were unstructured and variable; therefore, they proposed electronic handoffs for asynchronous transfer of information. Handoff documentation embedded within the electronic health record (EHR) facilitates easy and secure retrieval of pertinent information by various members of the care team [8]. Stein et al [20] showed that centralized electronic handoff documentation was utilized throughout the 24-hour period and not just during the usual shift change times. Handoffs often involve coordination between multiple individuals—each with varying levels of trainings, skills, and responsibility.

Handoffs that are a part of an EHR would facilitate remote and indirect supervision of the quality of handoff. Integration with the EHR would provide data security and enable controlled and accountable access to protected health information. Furthermore, it would facilitate root cause analysis in case of medical errors.

## Real-time Integration With EHR

Handoff EHR integration has been shown to aid in clinical decision-making and error-reporting [21]. Handoff information is typically updated at the end of the shift. EHR integration has been shown to improve communication and reduce errors [12,13]. The template must allow the incorporation of information by all the members of the interdisciplinary care team.

A real-time data feed from the EHR would enable the display of relevant up-to-date data (laboratory results, most recent vitals, medications, etc) on the same screen at the same time in which the handoff is being utilized, thereby facilitating better decisions.

## Direct Link Between Handoff Screens and Activity Screens

The health care team members should be able to undertake most tasks (eg, placing orders) from the handoff screen. Concurrently, they should be able to quickly navigate back and forth between the handoff screen and other parts of the EHR. The ease of navigation, the ability to review data for decision making, and the ability to implement the follow-up actions would encourage widespread adoption of the handoff.

## Availability in Mobile Devices

It is cumbersome for physicians and nurses to leave the task at hand, when paged or called by the patient, to log in to the EHR to find and view the information. Most nurses carry paper handoffs in their pockets during work, which adds an undue burden because loss of a handoff with patient data would be a significant HIPAA (Health Insurance Portability and Accountability Act) violation. Therefore, an increasing number of hospitals are providing nurses with HIPAA-secure mobile devices to facilitate their work.

The handoff should be adapted such that it is easily accessible, easy to view, and easy to navigate even on a mobile platform.

## Accessibility in Part to the Patient

Traditionally, handoffs occur between members of the patient care team. Granting all patients access to view a part of the handoff would keep them better informed about their plan of care.

Providing patients access to a part of the handoff would enable them to know their overall management plan, what was being planned for them on that day, and when they were approaching the expected day of discharge.

## Recommended Template

The first section should allow the medical team formulating the plan of care to summarize the chief reason for admission (Multimedia Appendix 1). This section should be followed by a brief overview of the medical management plan, including the pending evaluations and the actions to be taken in case of positive and negative laboratory test results. A projected course for the patient’s chief concern over the next 48 to 72 hours would ensure that the whole team is aware of the expected recovery course.

During the interdisciplinary team rounds in the morning, a daily task list that considers the patient’s goals and preferences should be formulated and added to the task list section of the handoff. The tasks should be timed for a specific time of the day so that all the interdisciplinary team members working in the following shifts are aware when the task is due to be completed. Timed interdisciplinary tasks as part of the handoff would not only allow the nursing team to execute the plan to ensure that the daily goals of care are met but also enable them to ensure that the pace is appropriate.

The expected date of discharge, the objective discharge criteria, as well as the stated goals of the patient/family regarding discharge should be a part of the handoff documentation to aid timely and safe transition of the patient to the next site of care. A free text box would allow the nurses, case managers, pharmacists, and other allied health care practitioners in different shifts to voice their concerns and contribute to the plan of care asynchronously, especially when the issue needs to be addressed during interdisciplinary rounds.

Every institute should adapt this template according to their individual needs. Physicians, residents, nurses, case managers, pharmacists, and other health care teams involved in caring for the patient should be taught how to use the handoff with clear expectations for updating it periodically. The handoff should be available within the EHR, pulling in real-time data from the EHR while allowing users to quickly navigate to action screens within the EHR. The same EHR should also be accessible via mobile devices. Hospitals should consider allowing patients to view a part, if not all, of the handoff.

## Appendix

Multimedia Appendix 1 Contents of a templated interdisciplinary handoff.

## Abbreviations

ACGME: Accreditation Council for Graduate Medical Education
CLER: Clinical Learning Environment Review
EHR: electronic health record
HIPAA: Health Insurance Portability and Accountability Act
SBAR: Situation, Background, Assessment, and Recommendation
