# Supplementary material for: Desirable Features of an Interdisciplinary Handoff
Source: JMIR Nurs. 2020 May 22;3(1):e18914. doi: 10.2196/18914 (PMC8279443; doi:10.2196/18914)
Supplement: Multimedia Appendix 1 [file nursing_v3i1e18914_app1.docx]

**Detailed individual patient page for hand-off**

Patient Name- Room Number- Age- Gender- MRN- Current Location- Code Status

Medical Team- Free Text- Reason for hospitalization- Main plan of care in brief – Discharge criteria

Nursing Team – Pharmacy Team – Case Management Team – Free Text- Needs from their viewpoints

Interdisciplinary 24 hour Task list for completion

Lines-Tubes-Drains, Allergies, Problem list

Vitals – Recent labs & diagnostics – Medications – Notes (Integrated- real time data updating these sections and ability to navigate to orders/ documentation sections from here)

**Summary patient list for handoff**

Patient name, room, Age, Gender

Main plan of care – Interdisciplinary task list

New results

Code Status
